# Diagnostic and Surgical Implications of Non-occlusive Mesenteric Ischemic Ileus Associated With Common Celiacomesenteric Trunk: A Case Report and Literature Review

**DOI:** 10.7759/cureus.54837

**Published:** 2024-02-24

**Authors:** Paul Joon Koo Choi, Jigyasha Pradhan, Sania Thite, Reshma Pydi, Gagan Sathya Prakash, Tiffany-Marie Golek, Sarah Moore, Ajay Shah, Hanasoge Girishkumar

**Affiliations:** 1 Surgery, BronxCare Health System, New York, USA; 2 Medicine, American University of the Caribbean School of Medicine, Cupecoy, SXM

**Keywords:** hepato-pancreato-biliary surgery, endovascular surgery, anatomic anomaly, dissection, acute mesenteric ischemia, thrombosis, aneurysm, celiomesenteric trunk, celiacomesenteric trunk

## Abstract

The celiacomesenteric trunk (CMT), an exceedingly rare anatomic variant uniting the celiac artery and superior mesenteric artery (SMA), holds significant clinical and surgical implications. Despite its rarity, understanding these implications is crucial for effective management. This report outlines the case of a 99-year-old female presenting with septic shock and abdominal pain, with imaging revealing an incidental CMT. This paper aims to elucidate the surgical implications associated with CMT through a comprehensive case review and literature search.

A 99-year-old female with multiple cardiovascular comorbidities presented with altered mental status and right lower quadrant abdominal pain. Upon arrival, the patient exhibited disorientation, an inability to follow commands, hypoxia, and hypotension. Significant laboratory findings included a white count of 20.6 x 10^9^/L, lactate of 6.1 mmol/L, glucose of 53 mg/dL, alanine transaminase (ALT)/aspartate aminotransferase (AST) of 186/336 U/L, and creatinine of 4.2 mg/dL. Immediate interventions involved high-flow oxygen, fluid resuscitation, intravenous antibiotics, and admission to the ICU for septic shock. A CT angiogram (CTA) revealed an incidental large common trunk comprising the celiac trunk and superior mesenteric artery (SMA). There was a high-grade stenosis at the origin of the SMA. However, all the vessels were widely patent distally, and acute mesenteric occlusion was ruled out. By day 12, the patient achieved clinical stability after conservative management and was discharged. Complications such as aneurysm, dissection, stenosis, thrombosis, or acute occlusion of a CMT may necessitate complex surgical interventions, including endovascular procedures or open hepatic surgery. Understanding these technical complexities is vital for avoiding surgical complications in critically ill patients.

## Introduction

Celiacomesenteric trunks (CMTs) make up less than 1% of all abdominal vascular anomalies, and their incidence varies from 0.4% to 2.7% [1,2]. Patients with this anatomic variation are often asymptomatic, and such a feature is discovered incidentally in cadaveric dissection or autopsies, during surgical procedures, imaging, and other diagnostic procedures [3]. It is well documented in the literature that CMTs may be associated with aneurysm, dissection, occlusion, and stenosis. Over the years, variations of CMTs have been documented, leading to the development of several classification systems aimed at better understanding these anatomical complexities [4-6].

Embryologically, the abdominal visceral arteries arise from the primitive dorsal aorta (DA) via four splanchnic roots, i.e., the left gastric artery, hepatic artery, splenic artery, and the superior mesenteric artery (SMA) [7]. These roots are joined together by a ventral longitudinal anastomosis (VLA), which is also known as Lang’s anastomosis [2,7,8]. Normally, a cleft forms in this anastomosis as it regresses between the third and fourth roots, isolating the celiac artery (CA) from the SMA [6,7,9,10]. This allows for the SMA to take off and originate approximately 1 cm below the CA at the L1 and L2 levels [7,9]. The persistence of the VLA higher than the fourth root may join CA branches with the SMA, and the regression of the first or fourth root may form a variant of CMT [7,9,11].

## Case presentation

We present the case of a 99-year-old female patient with multiple cardiovascular comorbidities, including hypertension, mitral and tricuspid regurgitations, chronic heart failure, atrial fibrillation and flutter, and pulmonary hypertension, who was brought into the ER by an ambulance due to altered mental status. It was also noted that the patient grimaced upon deep palpation of the abdomen, especially in the right lower quadrant. The patient's family reported that she experienced reduced oral intake since she had a fall two days ago. In the ER, the patient was noted to be disoriented, not following commands, hypoxic to 93% on high flow nasal cannula, hypotensive with systolic blood pressure (SBP) in the 50s and with a mean arterial pressure (MAP) of 63 mmHg, and bradycardic; she was immediately fluid resuscitated. Laboratory values were significant for a white count of 20.6 x 109/L, lactate of 6.1 mmol/L, glucose of 53 mg/dL, alanine transaminase (ALT)/aspartate aminotransferase (AST) of 186/336 U/L, and creatinine of 4.2 mg/dL. The patient was given intravenous antibiotics for suspected septic shock and transferred to the ICU for further management of the same.

**Figure 1:**
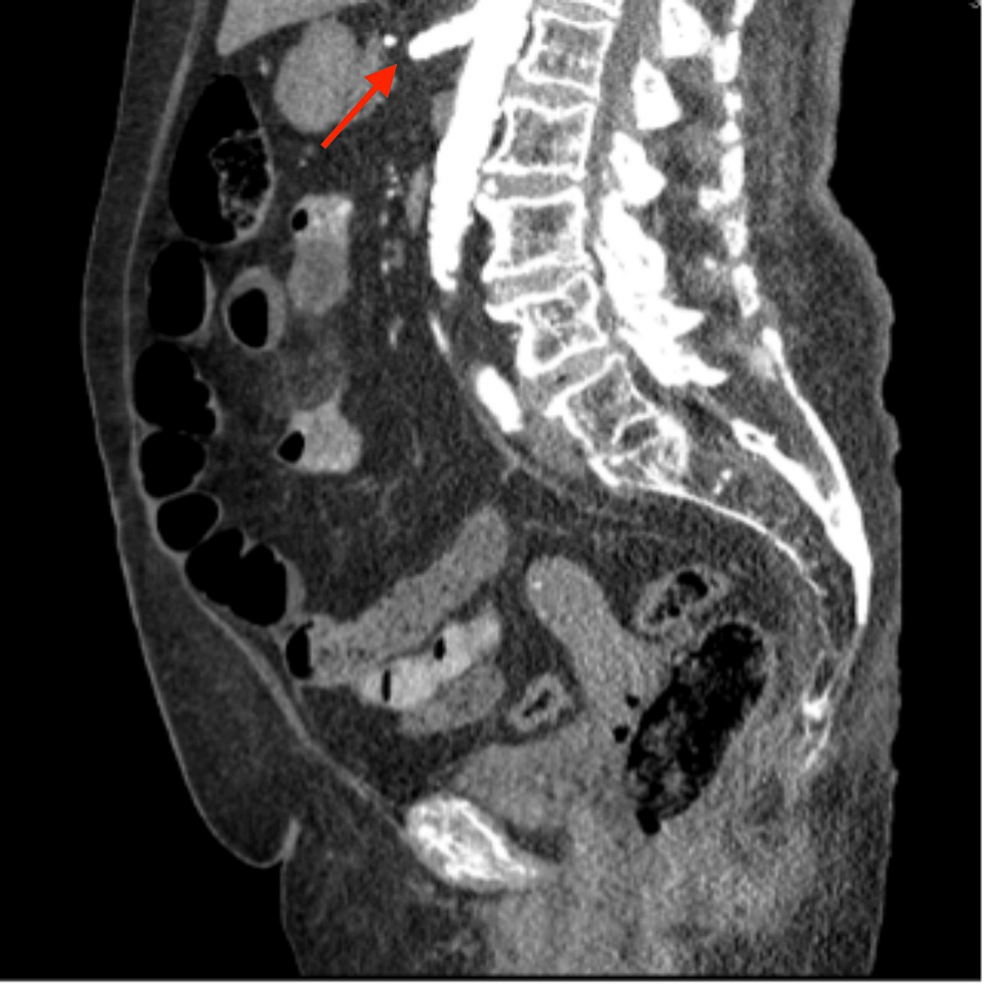
The CTA shows the CA and the SMA arising from a common trunk (red arrow) CTA: CT angiogram, CA: Celiac artery, SMA: Superior mesenteric artery

**Figure 2:**
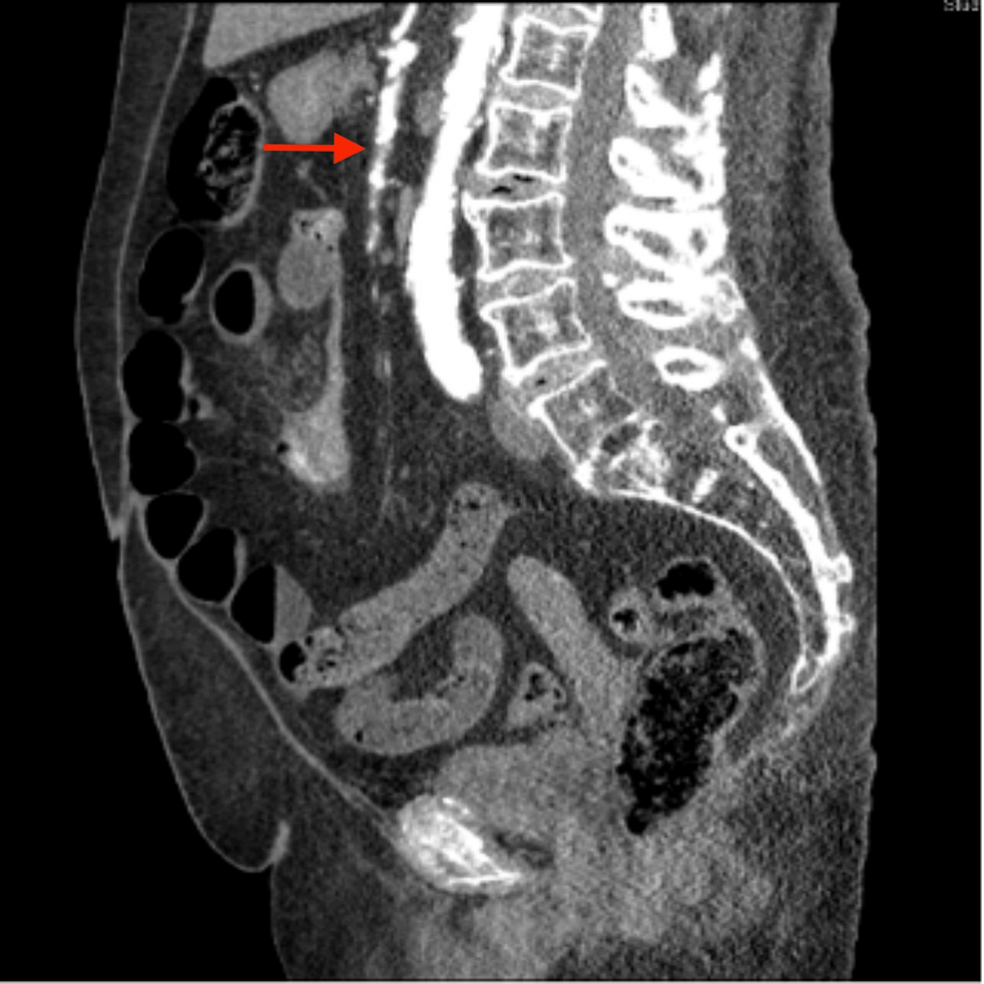
The red arrow depicts the SMA descending inferiorly directly from the common trunk SMA: Superior mesenteric artery

**Figure 3:**
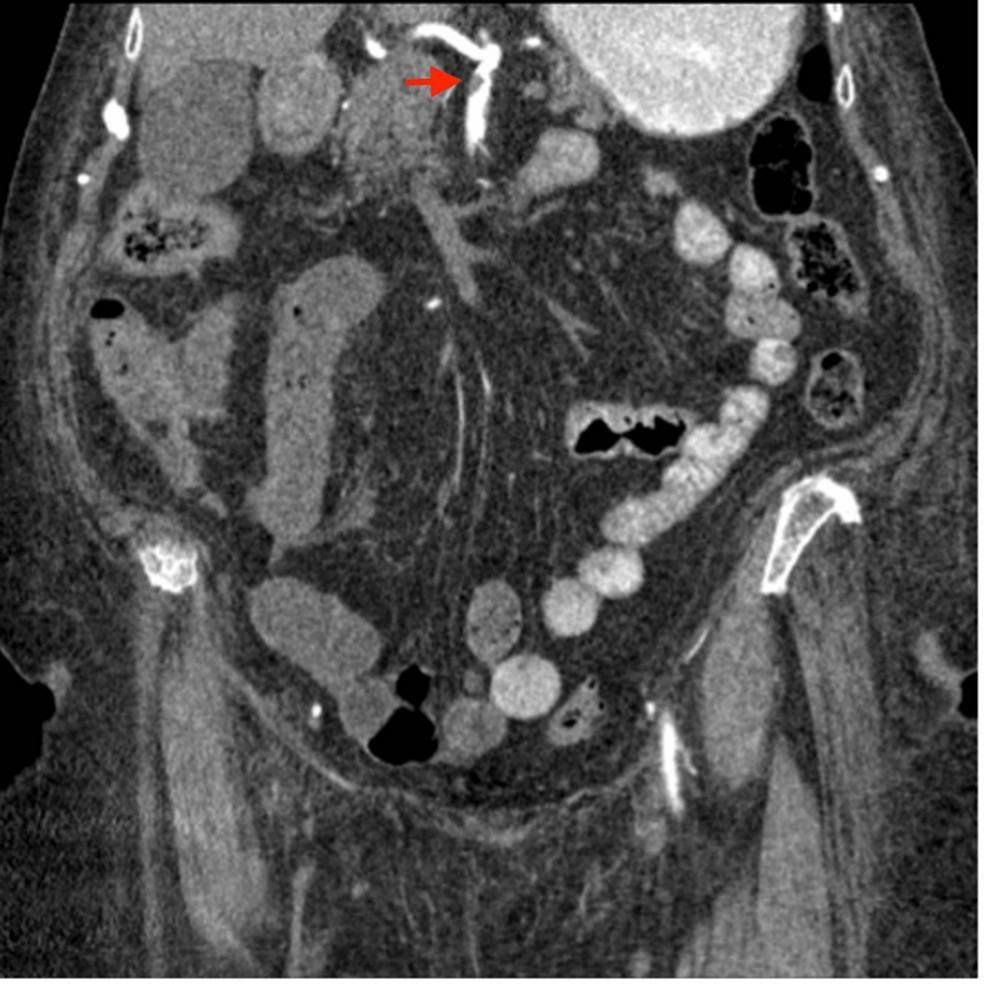
This CTA image illustrates a high-grade stenosis (red arrow) at the branching point of the SMA CTA: CT angiogram, SMA: Superior mesenteric artery

**Figure 4:**
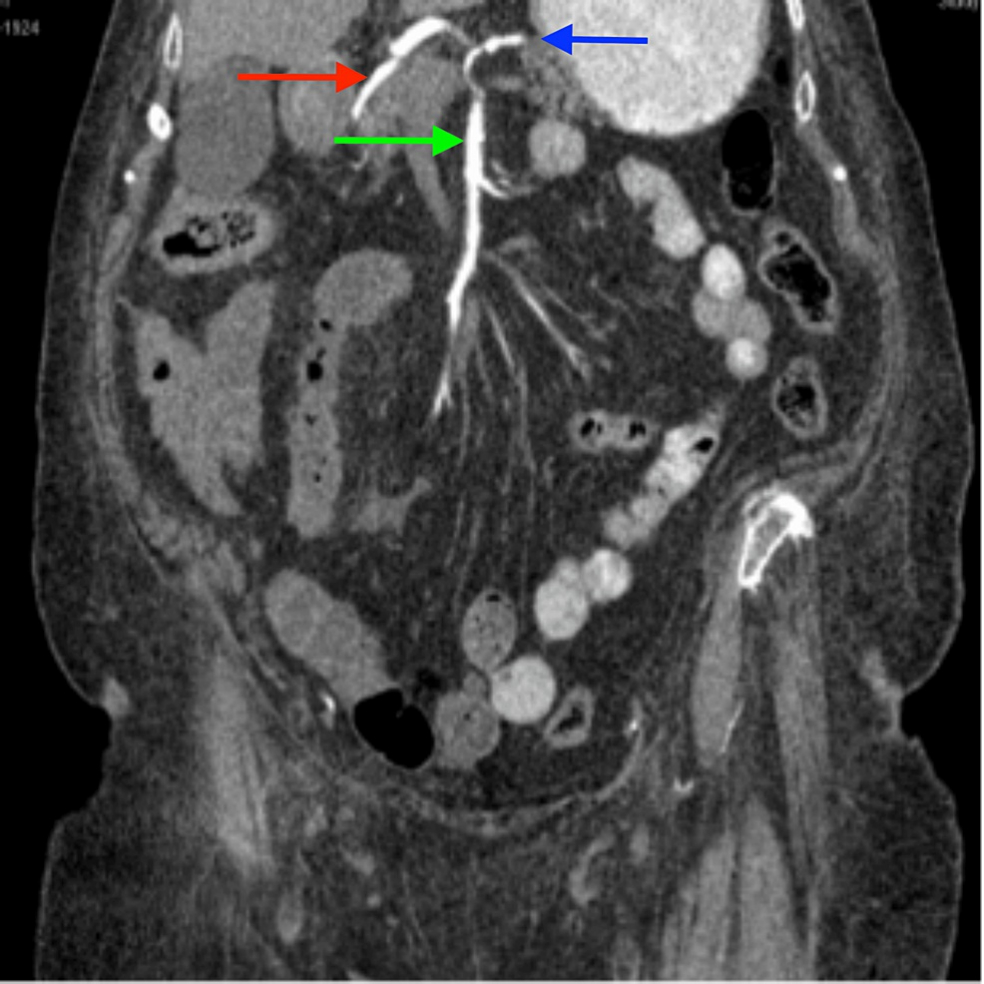
This coronal section displays the CHA (red arrow), LGA (blue arrow), and SMA (green arrow) originating from a common trunk CHA: Common hepatic artery, LGA: Left gastric artery, SMA: Superior mesenteric artery

A CT angiography (CTA) of the abdomen and pelvis showed an incidental finding of a large common trunk consistent with the celiac trunk and the SMA (Figures 1-3). There was a high-grade stenosis near and at the origin of the hepatic artery and SMA, respectively (Figure 4). However, provided all the branches were widely patent distally, there was low suspicion for mesenteric arterial occlusion. Also noted in the imaging were dilated loops of the small bowel with air-fluid levels, suggesting the possibility of an ileus. Several measures were taken in the ICU, and on day 2 of hospitalization, the patient’s lactate normalized to 1.5. On day 3, she became alert and oriented, followed commands, and denied any abdominal pain. On day 12, the patient experienced the return of normal bowel movements and was clinically stable for discharge.

## Discussion

Mesenteric ischemia

A CMT may be associated with aneurysm, dissection, occlusion, and stenosis. Any of these disease processes involving a single CMT can lead to devastating effects on abdominal organs. The loss of the CA-SMA collateral circulation leaves a large segment of the bowel with only one dominant vascular supply, making it prone to ischemic insult [7].

Acute mesenteric ischemia is sudden hypoperfusion of the small intestines secondary to occlusive or non-occlusive obstruction of the arterial inflow or cessation or reduction of venous outflow of the mesentery. Occlusive arterial disease is most commonly due to an acute embolism or thrombosis and most frequently involves the SMA. This can lead to lethal ischemia of the liver, stomach, duodenum, and small or large intestine. Lovisetto et al. described a case of an acute abdomen where a patient's CMT was acutely thrombosed, resulting in diffuse infarction involving the liver and spleen and extending from the stomach to the first third portion of the transverse colon [7].

In addition to acute mesenteric ischemia, patients with chronic mesenteric ischemia have also been described as having an underlying CMT phenomenon. For instance, Tasleem et al. documented a case involving an 81-year-old female patient presenting with mesenteric angina. The patient exhibited stenosis related to a CMT and was successfully treated using an endovascular technique [12]. In addition to endovascular approaches, open bypass graft placement has been utilized in certain cases to re-perfuse stenotic CMT in those presenting with recurrent mesenteric ischemia [13].

Our 99-year-old patient might have experienced transient non-occlusive mesenteric ischemia (NOMI) secondary to shock in the context of pre-existing CMT with high-grade thrombosis. The patient's susceptibility to NOMI may have been heightened by multiple cardiac comorbidities, such as heart failure and atrial fibrillation. Additionally, our CTA revealed dilated loops of the small bowel and air-fluid levels indicative of ileus, consistent with the patient's prolonged lack of bowel movement and suggestive of a possible ischemic insult to the small intestine. Ileus is a well-documented radiographic sign and complication of NOMI.

Celiac artery aneurysm

A celiac artery aneurysm (CAA) is very rare and even more uncommon in patients with CMT [14]. In general, visceral artery aneurysms are rare and found only in 0.01% to 0.2% of the population. An aneurysm of the CMT is extremely uncommon and occurs in only 0.25% of all visceral artery anomalies [14]. More than 20% of visceral artery aneurysms represent a medical emergency and more than 8% result in death due to rupture [14]. A CAA is known for its high rupture rate of 2% to 10%, with a mortality rate as high as 25%; hence, a surgical intervention is generally recommended if its sac is greater than 2 cm or for those who are pregnant or have portal hypertension, where heightened blood pressure may render greater risk of rupture [14]. Detroux et al. reported a case of a 5 cm calcified saccular aneurysm originating from a CMT bifurcation, which caused chronic epigastric pain persisting for 20 years [10]. Their patient was successfully managed via an open laparotomy followed by resection of the sac without vascular reconstruction [10].

Since CMT is near the pancreatic body, care should be taken not to injure the pancreas. Its distinctive anatomy renders endovascular surgery challenging and would often require open vascular reconstructive surgery. Oishi et al. described two cases of CMT aneurysms larger than 3 cm, in which they successfully utilized the great saphenous vein graft to replace and reconstruct the vascular anomalies [14]. However, with the advancement of imaging modalities and a more precise understanding of anatomy, endovascular coil embolization has also emerged as a viable option to address this unique disorder [15]. These advancements have made endovascular interventions more feasible than ever and offer alternative approaches for managing CMT aneurysms.

Implications of CMT in endovascular surgery

Although difficult, surgeons have been successful in deploying stent grafts in patients with a CA-SMA common trunk. For example, Fiorucci and Tsilimparis describe a three-vessel fenestrated endograft placement in a 70-year-old female patient who presented with an acute type B dissection [16]. Their report describes the use of CTA to plan the procedure. Malviya et al. performed CTAs on 110 patients and described variations in the vasculature of the upper gastrointestinal tract (GIT). They stressed that accurate vascular imaging was paramount in surgical planning for patients with upper GI issues [17]. This assertion was further reinforced by Fang et al., who described the increase in the use of endovascular interventions in complex vascular anatomies, including splenic artery aneurysms, in the background of a concomitant CMT [18]. Their approaches included endovascular embolization and covered stent deployment, which achieved a remarkable 95.5% technical success rate. Furthermore, a recent report by Ritenour and Mousa depicted the case of a 73-year-old female patient with acute occlusion of her CMT and proximal SMA who underwent successful endovascular repair with a stent graft via brachial access following meticulous anatomical analysis using CTA [19].

Vascular compression syndromes

Cases involving compression of the CMT by the median arcuate ligament (MAL) of the diaphragm have been reported, resulting in clinical symptoms such as epigastric pain, weight loss, and even mesenteric ischemia and death [20]. The anatomic proximity of a short CMT with MAL and the tight tendinous ring around the aortic opening may cause compression of the trunk, leading to postprandial periumbilical pain known as celiac trunk compression syndrome [20]. Braet et al. described a successful release of MAL via midline laparotomy, leading to symptomatic resolution and freeing the patient from chronic pain [21].

Nutcracker syndrome concerning a CMT has also been described in the literature. Peterson et al. reported that an autopsy study of a 91-year-old female patient who passed away from a cerebrovascular accident had an incidental finding of compression of the left renal vein by a narrow aortoceliac angle (ACA) created by a CMT, leading to engorgement of the left renal vein compared to its right counterpart [22]. Similarly, Al-Zoubi et al. documented a case of a 14-year-old boy presenting with chronic painless hematuria found to have nutcracker syndrome with an acute angle formed by a CMT anomaly [23]. Interestingly, an aneurysmal change of a CMT has also been described as causing this phenomenon [24].

The SMA syndrome is a duodenal obstruction secondary to acute angulation of the SMA-compressing part of the small intestine [25]. A 15-year-old who presented with colicky abdominal pain and bilious emesis was found to have a CT finding of partial duodenal obstruction by an acute angulation of a CMT concerning the abdominal aorta. For this patient, a duodenojejunostomy was performed, completely resolving his symptoms [26].

Implications of CMT in hepatobiliary surgeries

Anatomical variations in blood supply are pivotal considerations in planning open surgical and endoscopic cases [27]. Complex surgeries, including hepatobiliary, pancreatic, liver transplantation, and splenic procedures, demand thorough planning due to the intricate vascular anatomy involved [28,29]. The defined variations present possible management difficulties and risk mitigation of intraoperative and postoperative complications, such as bleeding and anastomotic leak [30].

Ataka et al. demonstrated the feasibility of a safe robotic distal pancreatectomy in a patient with pancreatic adenocarcinoma and an incidentally found CMT, highlighting the importance of utilizing accurate imaging modalities for surgical planning [27]. Similarly, Guglielmo et al. described a hepatic case where they performed vascular reconstruction using the aortic patch of the CMT and concluded the need for meticulous analysis of vascular anatomy before liver procurement or transplant [28].

It is intuitive to suggest that the rarity and severity of any novel anatomical variations, such as CMT, would substantially increase the risks of complications if not strategically mapped. The nature of these risks requires an understanding of these anatomical differences. A true appreciation for the anatomical variations in each patient is critical to successful outcomes and mitigating intraoperative and postoperative risks.

## Conclusions

With the advancement of imaging technologies, an increasing number of visceral artery anomalies and variations are being documented in the literature. Additionally, various pathologies have been associated with CMT, each presenting unique clinical manifestations. We presented the case of a 99-year-old patient who experienced transient NOMI secondary to shock in the context of pre-existing CMT with high-grade thrombosis. Her clinical course was complicated by postischemic ileus. Although our patient was discharged without any surgical interventions, it is important to understand the anatomic anomaly for potential life-saving interventions in critically ill patients.

The importance of precise imaging analysis and comprehensive surgical understanding has been underscored in the literature. Despite being an exceedingly rare anatomic variant, CMT bears significant clinical and surgical implications. Given the absence of universal management guidelines, understanding the technical and anatomical intricacies on a case-by-case basis is imperative for the successful management of such patients.
